# Using Models of Social Transmission to Examine the Spread of Longline Depredation Behavior among Sperm Whales in the Gulf of Alaska

**DOI:** 10.1371/journal.pone.0109079

**Published:** 2014-10-01

**Authors:** Zachary A. Schakner, Chris Lunsford, Janice Straley, Tomoharu Eguchi, Sarah L. Mesnick

**Affiliations:** 1 Department of Ecology and Evolutionary Biology, University of California Los Angeles, Los Angeles, CA, United States of America; 2 Alaska Fisheries Science Center, National Marine Fisheries Service, NOAA, Auke Bay Laboratories, Juneau, AK, United States of America; 3 University of Alaska Southeast, Sitka, AK, United States of America; 4 Southwest Fisheries Science Center, National Marine Fisheries Service, NOAA, La Jolla, CA, United States of America; Oregon Health and Science University, United States of America

## Abstract

Fishing, farming and ranching provide opportunities for predators to prey on resources concentrated by humans, a behavior termed depredation. In the Gulf of Alaska, observations of sperm whales depredating on fish caught on demersal longline gear dates back to the 1970s, with reported incidents increasing in the mid-1990s. Sperm whale depredation provides an opportunity to study the spread of a novel foraging behavior within a population. Data were collected during National Marine Fisheries Service longline surveys using demersal longline gear in waters off Alaska from 1998 to 2010. We evaluated whether observations of depredation fit predictions of social transmission by fitting the temporal and spatial spread of new observations of depredation to the Wave of Advance model. We found a significant, positive relationship between time and the distance of new observations from the diffusion center (r^2^ = 0.55, p-value  = 0.003). The data provide circumstantial evidence for social transmission of depredation. We discuss how changes in human activities in the region (fishing methods and regulations) have created a situation in which there is spatial-temporal overlap with foraging sperm whales, likely influencing when and how the behavior spread among the population.

## Introduction

Fishing, farming and ranching provide opportunities for predators to prey on resources concentrated by humans, a behavior termed depredation. In the oceans, increasing global fishing effort provides a multitude of opportunities for marine mammals to exploit prey caught on lines, in nets or aquaculture pens. The concentrated prey provides strong energetic incentives for predators presumably because of reduced costs of foraging. Attention to depredation by marine mammals is growing [1], [2]. It is observed in many fisheries, is economically costly and can cause injurious or lethal entanglement in gear [1], [2]. The global scope of depredation, along with observations of depredation rapidly spreading through some marine mammal populations raise questions about how new foraging behaviors arise and are transmitted and also have led to suggestion that the behavior may be socially transmitted [3], [4]. Quantifying the mechanisms by which behavioral traits spread in any wild population, however, is challenging because field studies rarely allow for reliably distinguishing between asocial and social mechanisms [5]. As stated in [6], these problems are exacerbated by the logistical challenges of accessibility and visibility inherent in studying marine mammals at sea.

Depredation of demersal longline catches is observed in high latitude feeding grounds frequented by adult male sperm whales (*Physeter macrocephalus*) in the eastern North Pacific, the North Atlantic and the Southern Ocean [7]. Early observations of sperm whale depredation of sablefish, (*Anoplopoma fimbria)* in the Gulf of Alaska are not well documented, but anecdotal accounts date back to mid-1970s [V. O'Connell 2012 pers. comm.]. In 1996, reports of depredation began to substantially increase in the region coincident with changes in fishery management that lengthened the fishing season [8], [9]. Longitudinal observations of sperm whale depredation provide an opportunity to examine the mechanisms underlying the diffusion of a new and complex behavior through a wild population. Studies of social learning in humans and terrestrial animals commonly utilize the assumption that socially transmitted behaviors are expected to show accelerated diffusion [10], [11], while traits acquired independently are not expected to arise in a spatially linked pattern nor to spread as quickly. Yet, while social transmission may increase the likelihood of an accelerating increase over time, the pattern can be generated by entirely asocial processes, and the shape of diffusion curves alone cannot be reliably interpreted as an indicator of social learning [12]. Newer methods, such as network based diffusion analysis, that take into account individual level information are more reliable [6], [13] yet are beyond the scope of many studies of wild animals. Using data collected during National Marine Fisheries Service (NMFS) assessment surveys for sablefish, we plot the occurrence of depredation over time to graphically illustrate the rate of behavioral transmission. We then investigate the spatial and temporal distribution of depredation using an indicator of social transmission, the Wave-of-Advance model.

The Wave-of-Advance model quantifies the relationship between first observation of a behavior and its spread over time [14], [15]. If a novel behavior is socially transmitted through a population, it is expected to have a diffusion center or geographical location where the innovation first arises, with new occurrences of the behavior progressively radiating outward through time. The Wave-of-Advance uses simple linear regression to test for a positive correlation between the distance a behavior has spread and time. This pattern was observed during the presumed social transmission of agriculture in Neolithic Europe [15], [16]. Quantitatively, social transmission is evidenced by a linear increase in the distances of new observations of the behavior from the diffusion center over time. Alternatively, if a behavior arises independently among multiple innovators, there would be scattered pattern with wide variation between space and time.

## Material and Methods

### (a) Data Collection

#### Survey background

**Figure 1:**
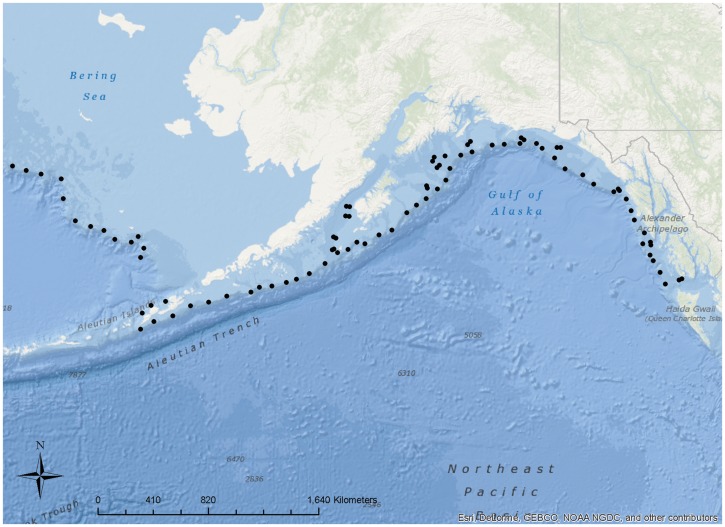
Survey area for sablefish assessment survey.

Data were collected during annual sablefish longline surveys conducted by the National Marine Fisheries Service from 1998 to 2010. These standardized, fishery-independent surveys cover the upper continental slope and selected gullies of the eastern Bering Sea, Aleutians Islands, and Gulf of Alaska. The surveys cover nearly all areas where adult sablefish are found and overlap commercial demersal longline fishing regions within the U.S. Exclusive Economic Zone. Sampling occurs annually in the summer and lasts three months. The survey follows a systematic design by placing stations that are 30–60 km apart at depths of 150 to 1000 m (Figure 1; Table S1 in File S1). Each year, approximately 90 stations were sampled. Beginning in 1998, observers began collecting data on depredation. Depredation was defined to occur when sperm whales were found near the vessel and adjacent to the longline during haulback (typically 100 m or less from the vessel) and when damaged sablefish were retrieved [16]. Characteristics of damaged sablefish include missing body parts, shredded tissue or lips remaining on hooks. At each station, the presence/absence of depredation was recorded. During a depredation event, the number of sperm whales present and the number of damaged sablefish were also recorded, but individual identification of the whales was not recorded.

### (b) Statistical analysis

We plotted the occurrence of depredation over time to illustrate the rate of behavioral transmission. The rate of behavioral transmission was modelled with the cumulative number of stations observed with depredation (y) as functions of time (x). Four functions were fit to the data: a function previously shown to indicate a constant rate [(linear (y  =  a_1_ + b_1_*x), decelerating rate [logarithmic (y  =  a_2_ + b_2_* log*x*)], and two functions indicating an accelerative rate of transmission [exponential (y  =  a_3_*e^(b3*x)^) and logistic (y  =  k/(1 +e^(a4 –r*x^))). We used an iterative least squares curve fitting procedure implemented by SigmaPlot (v.11). To compare fit of these models, we used Akaike's Information Criterion (AICc) for small sample sizes [17].

**Figure 2:**
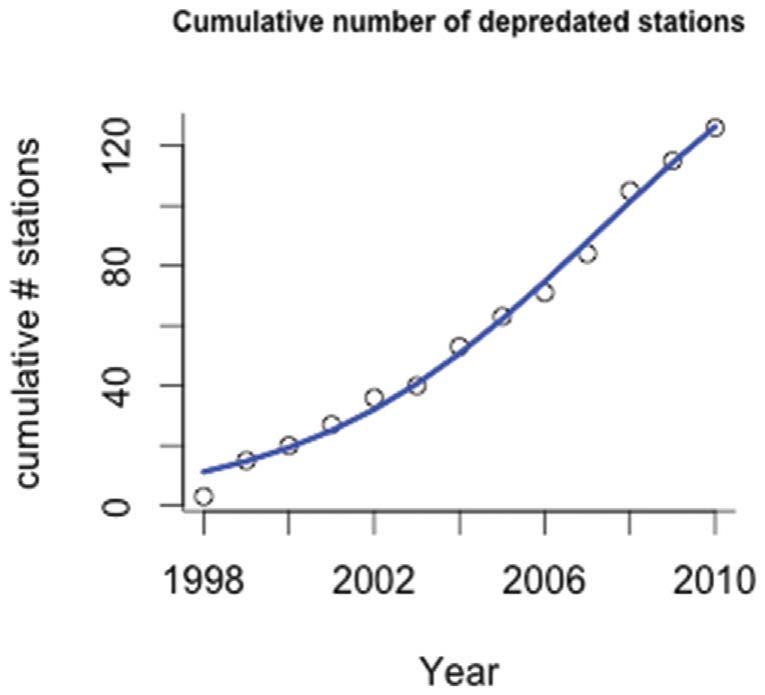
The cumulative number of stations with depredation over time with best fitting function (logistic).

We mapped geographic distribution of depredation through time to construct the Wave-of-Advance model. We estimated that the center of diffusion occurred in the location where depredation was observed in the first year of our study. Since depredation occurred at four stations in the Central Gulf (Figure 2a), we measured the midpoint of those stations for the diffusion center). With each successive survey year, the distance of the farthest new observation of depredation from the diffusion center was measured. These data were plotted with the distance from the center of diffusion as ordinate and survey year as abscissa and analyzed with a linear regression model in R [18] to determine if the relationship fit a linear spatial advance. We tested the null hypothesis that the slope is equal to zero. Under the null hypothesis, there is no relationship between the timing of new observations of depredation and their distance from the diffusion center, which would suggest the independent origin of the behavior. Statistical significance was tested at the type I error rate of 0.05.

## Results

Over the study period, depredation was observed on 126 occasions (Table S2 in File S1). The number of stations with depredation in a given year ranged from 4 (1998) to 22 (2008). The number of individuals present at stations with depredation ranged from 1–7 individuals (mean of 3.0). Depredation occurred only in the Gulf of Alaska and not along the Aleutian Islands or in the Bering Sea. The cumulative number of stations to observe depredation over time is presented in Figure 2. Over the study period, observations of depredation showed an accelerative pattern of increase over time. All four functions explained the variability in data (i.e., high r^2^ values), but the logistic function was the best model based on AICc (Table 1, Figure 2; see Figure S1 in File S1 for all functions).

**Table 1:** Rate of transmission analyses using Akaike Information Criterion (*best fit).

Model	# of Parameters	Parameter estimates	Uncorrected residual	r^2^	ΔAIC_c_	Σ*w_i_*
Logistic*	3	k = 182 r = 10.2, a_4_ = 3.5	124.7	.99	0	.995
Linear	2	a_1_ = 11.19 b_1_ = −10.04	382	.97	11.04	.003
Exponential	2	a_3_ = 15.4 b_3_ = .17	476.5	.97	14	.001
Logarithmic	2	a_2_ = −21.4, b_2_ = 46.4	3561	.79	40.14	.000

**Figure 3:**
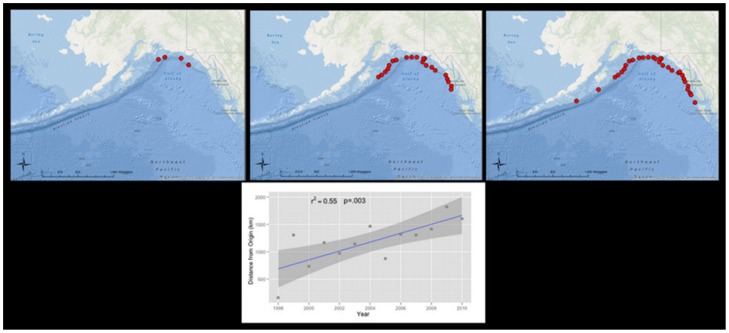
Spatial radiation of depredation and Wave-of-Advance model shows a positive correlation between time and the distance of new observations of depredation from origin (r^2^ = .55, p-value  = 0.003). Red dots represent stations to observe depredation. The panels, from left to right represent 1998, 1998–2003, and 1998–2010. The speed at which the behavior radiated is the slope of solid blue line, or 81.3±42.6 km/year (95% confidence interval). The solid blue line represents regression line and the gray shaded area is the 95% confidence interval for the line of best fit.

Depredation was first observed at four stations in 1998 and radiated from the West Yakutat region to the southeast and southwest during the 12 years (Figure 3). The Wave-of-Advance model shows a significant, positive relationship between time and the distance of new observations of depredation from the diffusion center (r^2^ = 0.55, p-value  = 0.003), thus we can reject the null hypothesis (slope = 0). The speed at which the behavior radiated is implied from the slope of the solid blue line, 81.3±47.9 km/year (95% confidence interval).

## Discussion

Exploration of the spatiotemporal spread of depredation behavior using both the rate of transmission and Wave-of-Advance analyses provides insights that neither method can alone. The observed spatiotemporal radiation of depredation in the Gulf of Alaska provides evidence, albeit circumstantial, for social transmission and provides context for further investigations as to why and how it evolved.

We acknowledge the limitations to both models which use stations as proxies for individuals yet enable us to provide standardized data throughout the study area. We are also unable to control for factors such as individual sperm whale movement or changes in commercial fishing effort that may provide alternative explanations for the observed spread of depredation. Male sperm whales are capable of travelling long distances [19], [20], are known to follow fishing vessels and some are likely to be repeat offenders among stations and years [20]. If the original innovator were a single whale, or a few individuals, that had learned to depredate commercial boats at the diffusion center and then expanded their range outward, this might explain the observed spatial pattern. This scenario, however, is unlikely because depredation continued to be observed at all intervening stations, including the diffusion center, during the course of all subsequent survey seasons. If there were changes in the distribution of sablefish, or of commercial fishing for sablefish in the Gulf of Alaska this might also influence the observed spatial radiation. If, for example, the distribution of fish or fishing effort increased outside the diffusion center in Central Gulf, there could potentially be more opportunities for individuals to acquire the behavior, which would be reflected in our survey as new stations with depredation. In addition, it is important to note that despite commercial fishing for sablefish throughout the study area, this study only observed depredation at research stations in the Gulf of Alaska. If the behavior was independently acquired, we would expect to see occurrences arise randomly anywhere in the study area, including in the Aleutians and Bering Sea. Further studies with the commercial fishing sector could help to illuminate these factors. In addition, further studies which relate the foraging preferences of sperm whales to fish availability may shed light on ecological factors influencing the behavior. For example, historic whaling records found that fish occurred in the stomachs of sperm whales more often in the Gulf of Alaska than along the Aleutians or in the Bering Sea [21] which may explain depredation being limited to the Gulf of Alaska.

The plot of the number of stations to experience depredation through time, and results of the function fitting exercise suggest an accelerated rate of increase in the occurrence of depredation among Gulf of Alaska sperm whales. We are cautious to infer social transmission from the transmission rate analysis alone, however, because of potential asocial explanations [12]. For instance, a decrease in neophobia (the avoidance response to novel stimuli), can influence transmission rates [22]. If the whales become less neophobic toward fishing gear over time, the rate at which they approach the fishing vessels might increase – resulting in an acceleratory curve without social transmission. In the case of sperm whale depredation, there is much room for the development of other methods, especially those that incorporate individual identification for investigating social transmission.

The conditions that gave rise to the original innovation are not well known, but the presence of longline fishing provides opportunities for individuals to learn to acquire prey off of the lines. Anecdotal accounts of longline depredation extend back to the 1970s, suggesting that this time period may represent a period of early innovation before the behavior became more common and widespread in the late 1990s. Beginning in 1984, the previously year-round sablefish season in the Gulf of Alaska was shortened, as short as 10 days in some years. In 1995, management shifted to an individual quota system, which extended the season to eight months. Fishing is now open March-November, overlapping spatially and temporally with the time when sperm whales naturally forage in the region. While our data are suggestive of a period of accelerated transmission, the lack of pre-1998 survey data is a major limitation and prevents examination of the events that may have caused the initial association or its expansion in any detail. In addition to changes in fishing season duration, other potential influences on the spread of depredation may be at play such as on-board fish processing and whether there is fishery offal (discard) in the water that may attract whales, changes in the abundance of fish stocks over time, and changes in the abundance of whales after the cessation of commercial whaling.

Despite a lack of behavioral data on individual whales at this time, the mechanisms (i.e., imitation, emulation, local enhancement) by which individuals might acquire behaviors consistent with depredation from conspecifics deserves further examination with other methods. While adult male sperm whales found on high latitude feeding grounds are generally thought to forage solitarily [19], our results also show that groups are more likely to be present during depredation events than solitary animals [Table S1 in File S1]. This observation is consistent with other studies in the region which have found that individually-identified males exhibit differing levels of association with vessels [23]. Depredation in groups was also more widespread in the later years of the study [Table S1 in File S1]. Aggregating around prey resources may facilitate social learning and may reveal an underlying male sociality, evidence of which is also observed in the existence of “bachelor schools” (loose aggregations of subadult males [19].) On-going studies to observe the behavior of depredating whales through satellite tagging and photo-identification studies are documenting male movements and may shed light on interactions among adult males. In addition, acoustic studies suggest individuals may eavesdrop for information on food aggregations [24]. Novel vocalization patterns produced during depredation [25] may convey cues used by nearby individuals and increase the probability of interaction through local or stimulus enhancement.

Human hunting, fishing and farming provides foraging opportunities for wildlife, resulting in the spread of novel foraging behaviors. The underlying transmission mechanisms have consequences for the rate and geographical spread of depredation. Social transmission can function as a multiplier in that new traits can spread more quickly and completely through a population than by individual acquisition. Knowledge of these mechanisms is important for management, which may find mitigation more tractable early on, before the behavior spreads through the population.

## Supporting Information

File S1 **Figure S1,** Cumulative number of stations fit with linear, exponential, logarithmic, and sigmoid functions. **Table S1,** Survey Coordinates. **Table S2,** Stations with depredation, 1998–2010. Station numbers are given along with the number of individuals observed at those stations (in parenthesis) for the years in which these data were recorded.
(DOCX)
