# Supplementary material for: Using Models of Social Transmission to Examine the Spread of Longline Depredation Behavior among Sperm Whales in the Gulf of Alaska
Source: PLoS One. 2014 Oct 1;9(10):e109079. doi: 10.1371/journal.pone.0109079 (PMC4182800; doi:10.1371/journal.pone.0109079)
Supplement: File S1 — Figure S1, Cumulative number of stations fit with linear, exponential, logarithmic, and sigmoid functions. Table S1, Survey Coordinates. Table S2, Stations with depredation, 1998–2010. Station numbers are given along with the number of individuals observed at those stations (in parenthesis) for the years in which these data were recorded. (DOCX) [file pone.0109079.s001.docx]

Table S1. Survey Coordinates

| Year | Survey | Station | Latitude | Longitude | Area |
| --- | --- | --- | --- | --- | --- |
| 2013 | United States | 1 | 58.778 | -177.575 | Bering Sea |
| 2013 | United States | 1 | 58.778 | -177.575 | Bering Sea |
| 2013 | United States | 1 | 58.778 | -177.575 | Bering Sea |
| 2013 | United States | 1 | 58.778 | -177.575 | Bering Sea |
| 2013 | United States | 1 | 58.778 | -177.575 | Bering Sea |
| 2013 | United States | 1 | 58.778 | -177.575 | Bering Sea |
| 2013 | United States | 1 | 58.778 | -177.575 | Bering Sea |
| 2013 | United States | 2 | 58.62 | -176.642 | Bering Sea |
| 2013 | United States | 2 | 58.62 | -176.642 | Bering Sea |
| 2013 | United States | 2 | 58.62 | -176.642 | Bering Sea |
| 2013 | United States | 2 | 58.62 | -176.642 | Bering Sea |
| 2013 | United States | 2 | 58.62 | -176.642 | Bering Sea |
| 2013 | United States | 2 | 58.62 | -176.642 | Bering Sea |
| 2013 | United States | 2 | 58.62 | -176.642 | Bering Sea |
| 2013 | United States | 4 | 58.497 | -175.67 | Bering Sea |
| 2013 | United States | 4 | 58.497 | -175.67 | Bering Sea |
| 2013 | United States | 4 | 58.497 | -175.67 | Bering Sea |
| 2013 | United States | 4 | 58.497 | -175.67 | Bering Sea |
| 2013 | United States | 4 | 58.497 | -175.67 | Bering Sea |
| 2013 | United States | 4 | 58.497 | -175.67 | Bering Sea |
| 2013 | United States | 4 | 58.497 | -175.67 | Bering Sea |
| 2013 | United States | 6 | 58.333 | -174.312 | Bering Sea |
| 2013 | United States | 6 | 58.333 | -174.312 | Bering Sea |
| 2013 | United States | 6 | 58.333 | -174.312 | Bering Sea |
| 2013 | United States | 6 | 58.333 | -174.312 | Bering Sea |
| 2013 | United States | 6 | 58.333 | -174.312 | Bering Sea |
| 2013 | United States | 6 | 58.333 | -174.312 | Bering Sea |
| 2013 | United States | 6 | 58.333 | -174.312 | Bering Sea |
| 2013 | United States | 8 | 57.628 | -174.165 | Bering Sea |
| 2013 | United States | 8 | 57.628 | -174.165 | Bering Sea |
| 2013 | United States | 8 | 57.628 | -174.165 | Bering Sea |
| 2013 | United States | 8 | 57.628 | -174.165 | Bering Sea |
| 2013 | United States | 8 | 57.628 | -174.165 | Bering Sea |
| 2013 | United States | 8 | 57.628 | -174.165 | Bering Sea |
| 2013 | United States | 8 | 57.628 | -174.165 | Bering Sea |
| 2013 | United States | 10 | 56.828 | -173.378 | Bering Sea |
| 2013 | United States | 10 | 56.828 | -173.378 | Bering Sea |
| 2013 | United States | 10 | 56.828 | -173.378 | Bering Sea |
| 2013 | United States | 10 | 56.828 | -173.378 | Bering Sea |
| 2013 | United States | 10 | 56.828 | -173.378 | Bering Sea |
| 2013 | United States | 10 | 56.828 | -173.378 | Bering Sea |
| 2013 | United States | 10 | 56.828 | -173.378 | Bering Sea |
| 2013 | United States | 12 | 56.627 | -172.353 | Bering Sea |
| 2013 | United States | 12 | 56.627 | -172.353 | Bering Sea |
| 2013 | United States | 12 | 56.627 | -172.353 | Bering Sea |
| 2013 | United States | 12 | 56.627 | -172.353 | Bering Sea |
| 2013 | United States | 12 | 56.627 | -172.353 | Bering Sea |
| 2013 | United States | 12 | 56.627 | -172.353 | Bering Sea |
| 2013 | United States | 12 | 56.627 | -172.353 | Bering Sea |
| 2013 | United States | 13 | 56.467 | -171.452 | Bering Sea |
| 2013 | United States | 13 | 56.467 | -171.452 | Bering Sea |
| 2013 | United States | 13 | 56.467 | -171.452 | Bering Sea |
| 2013 | United States | 13 | 56.467 | -171.452 | Bering Sea |
| 2013 | United States | 13 | 56.467 | -171.452 | Bering Sea |
| 2013 | United States | 13 | 56.467 | -171.452 | Bering Sea |
| 2013 | United States | 13 | 56.467 | -171.452 | Bering Sea |
| 2013 | United States | 15 | 56.147 | -170.732 | Bering Sea |
| 2013 | United States | 15 | 56.147 | -170.732 | Bering Sea |
| 2013 | United States | 15 | 56.147 | -170.732 | Bering Sea |
| 2013 | United States | 15 | 56.147 | -170.732 | Bering Sea |
| 2013 | United States | 15 | 56.147 | -170.732 | Bering Sea |
| 2013 | United States | 15 | 56.147 | -170.732 | Bering Sea |
| 2013 | United States | 15 | 56.147 | -170.732 | Bering Sea |
| 2013 | United States | 17 | 56.038 | -169.618 | Bering Sea |
| 2013 | United States | 17 | 56.038 | -169.618 | Bering Sea |
| 2013 | United States | 17 | 56.038 | -169.618 | Bering Sea |
| 2013 | United States | 17 | 56.038 | -169.618 | Bering Sea |
| 2013 | United States | 17 | 56.038 | -169.618 | Bering Sea |
| 2013 | United States | 17 | 56.038 | -169.618 | Bering Sea |
| 2013 | United States | 17 | 56.038 | -169.618 | Bering Sea |
| 2013 | United States | 18 | 56.243 | -169.172 | Bering Sea |
| 2013 | United States | 18 | 56.243 | -169.172 | Bering Sea |
| 2013 | United States | 18 | 56.243 | -169.172 | Bering Sea |
| 2013 | United States | 18 | 56.243 | -169.172 | Bering Sea |
| 2013 | United States | 18 | 56.243 | -169.172 | Bering Sea |
| 2013 | United States | 18 | 56.243 | -169.172 | Bering Sea |
| 2013 | United States | 18 | 56.243 | -169.172 | Bering Sea |
| 2013 | United States | 20 | 55.808 | -168.802 | Bering Sea |
| 2013 | United States | 20 | 55.808 | -168.802 | Bering Sea |
| 2013 | United States | 20 | 55.808 | -168.802 | Bering Sea |
| 2013 | United States | 20 | 55.808 | -168.802 | Bering Sea |
| 2013 | United States | 20 | 55.808 | -168.802 | Bering Sea |
| 2013 | United States | 20 | 55.808 | -168.802 | Bering Sea |
| 2013 | United States | 20 | 55.808 | -168.802 | Bering Sea |
| 2013 | United States | 22 | 55.458 | -168.998 | Bering Sea |
| 2013 | United States | 22 | 55.458 | -168.998 | Bering Sea |
| 2013 | United States | 22 | 55.458 | -168.998 | Bering Sea |
| 2013 | United States | 22 | 55.458 | -168.998 | Bering Sea |
| 2013 | United States | 22 | 55.458 | -168.998 | Bering Sea |
| 2013 | United States | 22 | 55.458 | -168.998 | Bering Sea |
| 2013 | United States | 22 | 55.458 | -168.998 | Bering Sea |
| 2013 | United States | 32 | 53.772 | -167.33 | Bering Sea |
| 2013 | United States | 32 | 53.772 | -167.33 | Bering Sea |
| 2013 | United States | 32 | 53.772 | -167.33 | Bering Sea |
| 2013 | United States | 32 | 53.772 | -167.33 | Bering Sea |
| 2013 | United States | 32 | 53.772 | -167.33 | Bering Sea |
| 2013 | United States | 32 | 53.772 | -167.33 | Bering Sea |
| 2013 | United States | 32 | 53.772 | -167.33 | Bering Sea |
| 2013 | United States | 33 | 53.612 | -168.298 | Bering Sea |
| 2013 | United States | 33 | 53.612 | -168.298 | Bering Sea |
| 2013 | United States | 33 | 53.612 | -168.298 | Bering Sea |
| 2013 | United States | 33 | 53.612 | -168.298 | Bering Sea |
| 2013 | United States | 33 | 53.612 | -168.298 | Bering Sea |
| 2013 | United States | 33 | 53.612 | -168.298 | Bering Sea |
| 2013 | United States | 33 | 53.612 | -168.298 | Bering Sea |
| 2013 | United States | 34 | 53.305 | -168.898 | Bering Sea |
| 2013 | United States | 34 | 53.305 | -168.898 | Bering Sea |
| 2013 | United States | 34 | 53.305 | -168.898 | Bering Sea |
| 2013 | United States | 34 | 53.305 | -168.898 | Bering Sea |
| 2013 | United States | 34 | 53.305 | -168.898 | Bering Sea |
| 2013 | United States | 34 | 53.305 | -168.898 | Bering Sea |
| 2013 | United States | 62 | 52.66 | -169.003 | Western Gulf of Alaska |
| 2013 | United States | 62 | 52.66 | -169.003 | Western Gulf of Alaska |
| 2013 | United States | 62 | 52.66 | -169.003 | Western Gulf of Alaska |
| 2013 | United States | 62 | 52.66 | -169.003 | Western Gulf of Alaska |
| 2013 | United States | 62 | 52.66 | -169.003 | Western Gulf of Alaska |
| 2013 | United States | 62 | 52.66 | -169.003 | Western Gulf of Alaska |
| 2013 | United States | 62 | 52.66 | -169.003 | Western Gulf of Alaska |
| 2013 | United States | 63 | 52.965 | -168.135 | Western Gulf of Alaska |
| 2013 | United States | 63 | 52.965 | -168.135 | Western Gulf of Alaska |
| 2013 | United States | 63 | 52.965 | -168.135 | Western Gulf of Alaska |
| 2013 | United States | 63 | 52.965 | -168.135 | Western Gulf of Alaska |
| 2013 | United States | 63 | 52.965 | -168.135 | Western Gulf of Alaska |
| 2013 | United States | 63 | 52.965 | -168.135 | Western Gulf of Alaska |
| 2013 | United States | 63 | 52.965 | -168.135 | Western Gulf of Alaska |
| 2013 | United States | 64 | 53.192 | -166.855 | Western Gulf of Alaska |
| 2013 | United States | 64 | 53.192 | -166.855 | Western Gulf of Alaska |
| 2013 | United States | 64 | 53.192 | -166.855 | Western Gulf of Alaska |
| 2013 | United States | 64 | 53.192 | -166.855 | Western Gulf of Alaska |
| 2013 | United States | 64 | 53.192 | -166.855 | Western Gulf of Alaska |
| 2013 | United States | 64 | 53.192 | -166.855 | Western Gulf of Alaska |
| 2013 | United States | 64 | 53.192 | -166.855 | Western Gulf of Alaska |
| 2013 | United States | 65 | 53.583 | -165.685 | Western Gulf of Alaska |
| 2013 | United States | 65 | 53.583 | -165.685 | Western Gulf of Alaska |
| 2013 | United States | 65 | 53.583 | -165.685 | Western Gulf of Alaska |
| 2013 | United States | 65 | 53.583 | -165.685 | Western Gulf of Alaska |
| 2013 | United States | 65 | 53.583 | -165.685 | Western Gulf of Alaska |
| 2013 | United States | 65 | 53.583 | -165.685 | Western Gulf of Alaska |
| 2013 | United States | 65 | 53.583 | -165.685 | Western Gulf of Alaska |
| 2013 | United States | 66 | 53.737 | -164.468 | Western Gulf of Alaska |
| 2013 | United States | 66 | 53.737 | -164.468 | Western Gulf of Alaska |
| 2013 | United States | 66 | 53.737 | -164.468 | Western Gulf of Alaska |
| 2013 | United States | 66 | 53.737 | -164.468 | Western Gulf of Alaska |
| 2013 | United States | 66 | 53.737 | -164.468 | Western Gulf of Alaska |
| 2013 | United States | 66 | 53.737 | -164.468 | Western Gulf of Alaska |
| 2013 | United States | 66 | 53.737 | -164.468 | Western Gulf of Alaska |
| 2013 | United States | 67 | 53.97 | -163.263 | Western Gulf of Alaska |
| 2013 | United States | 67 | 53.97 | -163.263 | Western Gulf of Alaska |
| 2013 | United States | 67 | 53.97 | -163.263 | Western Gulf of Alaska |
| 2013 | United States | 67 | 53.97 | -163.263 | Western Gulf of Alaska |
| 2013 | United States | 67 | 53.97 | -163.263 | Western Gulf of Alaska |
| 2013 | United States | 67 | 53.97 | -163.263 | Western Gulf of Alaska |
| 2013 | United States | 67 | 53.97 | -163.263 | Western Gulf of Alaska |
| 2013 | United States | 68 | 54.133 | -161.637 | Western Gulf of Alaska |
| 2013 | United States | 68 | 54.133 | -161.637 | Western Gulf of Alaska |
| 2013 | United States | 68 | 54.133 | -161.637 | Western Gulf of Alaska |
| 2013 | United States | 68 | 54.133 | -161.637 | Western Gulf of Alaska |
| 2013 | United States | 68 | 54.133 | -161.637 | Western Gulf of Alaska |
| 2013 | United States | 68 | 54.133 | -161.637 | Western Gulf of Alaska |
| 2013 | United States | 68 | 54.133 | -161.637 | Western Gulf of Alaska |
| 2013 | United States | 69 | 54.315 | -161.06 | Western Gulf of Alaska |
| 2013 | United States | 69 | 54.315 | -161.06 | Western Gulf of Alaska |
| 2013 | United States | 69 | 54.315 | -161.06 | Western Gulf of Alaska |
| 2013 | United States | 69 | 54.315 | -161.06 | Western Gulf of Alaska |
| 2013 | United States | 69 | 54.315 | -161.06 | Western Gulf of Alaska |
| 2013 | United States | 69 | 54.315 | -161.06 | Western Gulf of Alaska |
| 2013 | United States | 69 | 54.315 | -161.06 | Western Gulf of Alaska |
| 2013 | United States | 70 | 54.365 | -160.235 | Western Gulf of Alaska |
| 2013 | United States | 70 | 54.365 | -160.235 | Western Gulf of Alaska |
| 2013 | United States | 70 | 54.365 | -160.235 | Western Gulf of Alaska |
| 2013 | United States | 70 | 54.365 | -160.235 | Western Gulf of Alaska |
| 2013 | United States | 70 | 54.365 | -160.235 | Western Gulf of Alaska |
| 2013 | United States | 70 | 54.365 | -160.235 | Western Gulf of Alaska |
| 2013 | United States | 70 | 54.365 | -160.235 | Western Gulf of Alaska |
| 2013 | United States | 71 | 54.51 | -159.255 | Western Gulf of Alaska |
| 2013 | United States | 71 | 54.51 | -159.255 | Western Gulf of Alaska |
| 2013 | United States | 71 | 54.51 | -159.255 | Western Gulf of Alaska |
| 2013 | United States | 71 | 54.51 | -159.255 | Western Gulf of Alaska |
| 2013 | United States | 71 | 54.51 | -159.255 | Western Gulf of Alaska |
| 2013 | United States | 71 | 54.51 | -159.255 | Western Gulf of Alaska |
| 2013 | United States | 71 | 54.51 | -159.255 | Western Gulf of Alaska |
| 2013 | United States | 72 | 54.633 | -158.58 | Central Gulf of Alaska |
| 2013 | United States | 72 | 54.633 | -158.58 | Central Gulf of Alaska |
| 2013 | United States | 72 | 54.633 | -158.58 | Central Gulf of Alaska |
| 2013 | United States | 72 | 54.633 | -158.58 | Central Gulf of Alaska |
| 2013 | United States | 72 | 54.633 | -158.58 | Central Gulf of Alaska |
| 2013 | United States | 72 | 54.633 | -158.58 | Central Gulf of Alaska |
| 2013 | United States | 72 | 54.633 | -158.58 | Central Gulf of Alaska |
| 2013 | United States | 73 | 54.852 | -157.737 | Central Gulf of Alaska |
| 2013 | United States | 73 | 54.852 | -157.737 | Central Gulf of Alaska |
| 2013 | United States | 73 | 54.852 | -157.737 | Central Gulf of Alaska |
| 2013 | United States | 73 | 54.852 | -157.737 | Central Gulf of Alaska |
| 2013 | United States | 73 | 54.852 | -157.737 | Central Gulf of Alaska |
| 2013 | United States | 73 | 54.852 | -157.737 | Central Gulf of Alaska |
| 2013 | United States | 73 | 54.852 | -157.737 | Central Gulf of Alaska |
| 2013 | United States | 74 | 55.24 | -156.673 | Central Gulf of Alaska |
| 2013 | United States | 74 | 55.24 | -156.673 | Central Gulf of Alaska |
| 2013 | United States | 74 | 55.24 | -156.673 | Central Gulf of Alaska |
| 2013 | United States | 74 | 55.24 | -156.673 | Central Gulf of Alaska |
| 2013 | United States | 74 | 55.24 | -156.673 | Central Gulf of Alaska |
| 2013 | United States | 74 | 55.24 | -156.673 | Central Gulf of Alaska |
| 2013 | United States | 74 | 55.24 | -156.673 | Central Gulf of Alaska |
| 2013 | United States | 75 | 55.642 | -155.848 | Central Gulf of Alaska |
| 2013 | United States | 75 | 55.642 | -155.848 | Central Gulf of Alaska |
| 2013 | United States | 75 | 55.642 | -155.848 | Central Gulf of Alaska |
| 2013 | United States | 75 | 55.642 | -155.848 | Central Gulf of Alaska |
| 2013 | United States | 75 | 55.642 | -155.848 | Central Gulf of Alaska |
| 2013 | United States | 75 | 55.642 | -155.848 | Central Gulf of Alaska |
| 2013 | United States | 76 | 55.767 | -155.138 | Central Gulf of Alaska |
| 2013 | United States | 76 | 55.767 | -155.138 | Central Gulf of Alaska |
| 2013 | United States | 76 | 55.767 | -155.138 | Central Gulf of Alaska |
| 2013 | United States | 76 | 55.767 | -155.138 | Central Gulf of Alaska |
| 2013 | United States | 76 | 55.767 | -155.138 | Central Gulf of Alaska |
| 2013 | United States | 76 | 55.767 | -155.138 | Central Gulf of Alaska |
| 2013 | United States | 76 | 55.767 | -155.138 | Central Gulf of Alaska |
| 2013 | United States | 77 | 56.042 | -154.567 | Central Gulf of Alaska |
| 2013 | United States | 77 | 56.042 | -154.567 | Central Gulf of Alaska |
| 2013 | United States | 77 | 56.042 | -154.567 | Central Gulf of Alaska |
| 2013 | United States | 77 | 56.042 | -154.567 | Central Gulf of Alaska |
| 2013 | United States | 77 | 56.042 | -154.567 | Central Gulf of Alaska |
| 2013 | United States | 77 | 56.042 | -154.567 | Central Gulf of Alaska |
| 2013 | United States | 77 | 56.042 | -154.567 | Central Gulf of Alaska |
| 2013 | United States | 78 | 55.973 | -154.022 | Central Gulf of Alaska |
| 2013 | United States | 78 | 55.973 | -154.022 | Central Gulf of Alaska |
| 2013 | United States | 78 | 55.973 | -154.022 | Central Gulf of Alaska |
| 2013 | United States | 78 | 55.973 | -154.022 | Central Gulf of Alaska |
| 2013 | United States | 78 | 55.973 | -154.022 | Central Gulf of Alaska |
| 2013 | United States | 78 | 55.973 | -154.022 | Central Gulf of Alaska |
| 2013 | United States | 78 | 55.973 | -154.022 | Central Gulf of Alaska |
| 2013 | United States | 79 | 56.303 | -153.077 | Central Gulf of Alaska |
| 2013 | United States | 79 | 56.303 | -153.077 | Central Gulf of Alaska |
| 2013 | United States | 79 | 56.303 | -153.077 | Central Gulf of Alaska |
| 2013 | United States | 79 | 56.303 | -153.077 | Central Gulf of Alaska |
| 2013 | United States | 79 | 56.303 | -153.077 | Central Gulf of Alaska |
| 2013 | United States | 79 | 56.303 | -153.077 | Central Gulf of Alaska |
| 2013 | United States | 80 | 56.485 | -152.213 | Central Gulf of Alaska |
| 2013 | United States | 80 | 56.485 | -152.213 | Central Gulf of Alaska |
| 2013 | United States | 80 | 56.485 | -152.213 | Central Gulf of Alaska |
| 2013 | United States | 80 | 56.485 | -152.213 | Central Gulf of Alaska |
| 2013 | United States | 80 | 56.485 | -152.213 | Central Gulf of Alaska |
| 2013 | United States | 80 | 56.485 | -152.213 | Central Gulf of Alaska |
| 2013 | United States | 80 | 56.485 | -152.213 | Central Gulf of Alaska |
| 2013 | United States | 81 | 57.118 | -151.222 | Central Gulf of Alaska |
| 2013 | United States | 81 | 57.118 | -151.222 | Central Gulf of Alaska |
| 2013 | United States | 81 | 57.118 | -151.222 | Central Gulf of Alaska |
| 2013 | United States | 81 | 57.118 | -151.222 | Central Gulf of Alaska |
| 2013 | United States | 81 | 57.118 | -151.222 | Central Gulf of Alaska |
| 2013 | United States | 81 | 57.118 | -151.222 | Central Gulf of Alaska |
| 2013 | United States | 82 | 57.402 | -150.573 | Central Gulf of Alaska |
| 2013 | United States | 82 | 57.402 | -150.573 | Central Gulf of Alaska |
| 2013 | United States | 82 | 57.402 | -150.573 | Central Gulf of Alaska |
| 2013 | United States | 82 | 57.402 | -150.573 | Central Gulf of Alaska |
| 2013 | United States | 82 | 57.402 | -150.573 | Central Gulf of Alaska |
| 2013 | United States | 82 | 57.402 | -150.573 | Central Gulf of Alaska |
| 2013 | United States | 83 | 57.632 | -149.917 | Central Gulf of Alaska |
| 2013 | United States | 83 | 57.632 | -149.917 | Central Gulf of Alaska |
| 2013 | United States | 83 | 57.632 | -149.917 | Central Gulf of Alaska |
| 2013 | United States | 83 | 57.632 | -149.917 | Central Gulf of Alaska |
| 2013 | United States | 83 | 57.632 | -149.917 | Central Gulf of Alaska |
| 2013 | United States | 83 | 57.632 | -149.917 | Central Gulf of Alaska |
| 2013 | United States | 84 | 57.972 | -149.167 | Central Gulf of Alaska |
| 2013 | United States | 84 | 57.972 | -149.167 | Central Gulf of Alaska |
| 2013 | United States | 84 | 57.972 | -149.167 | Central Gulf of Alaska |
| 2013 | United States | 84 | 57.972 | -149.167 | Central Gulf of Alaska |
| 2013 | United States | 84 | 57.972 | -149.167 | Central Gulf of Alaska |
| 2013 | United States | 84 | 57.972 | -149.167 | Central Gulf of Alaska |
| 2013 | United States | 84 | 57.972 | -149.167 | Central Gulf of Alaska |
| 2013 | United States | 85 | 58.293 | -148.617 | Central Gulf of Alaska |
| 2013 | United States | 85 | 58.293 | -148.617 | Central Gulf of Alaska |
| 2013 | United States | 85 | 58.293 | -148.617 | Central Gulf of Alaska |
| 2013 | United States | 85 | 58.293 | -148.617 | Central Gulf of Alaska |
| 2013 | United States | 85 | 58.293 | -148.617 | Central Gulf of Alaska |
| 2013 | United States | 85 | 58.293 | -148.617 | Central Gulf of Alaska |
| 2013 | United States | 85 | 58.293 | -148.617 | Central Gulf of Alaska |
| 2013 | United States | 86 | 58.688 | -148.34 | Central Gulf of Alaska |
| 2013 | United States | 86 | 58.688 | -148.34 | Central Gulf of Alaska |
| 2013 | United States | 86 | 58.688 | -148.34 | Central Gulf of Alaska |
| 2013 | United States | 86 | 58.688 | -148.34 | Central Gulf of Alaska |
| 2013 | United States | 86 | 58.688 | -148.34 | Central Gulf of Alaska |
| 2013 | United States | 86 | 58.688 | -148.34 | Central Gulf of Alaska |
| 2013 | United States | 87 | 59.127 | -148.65 | Central Gulf of Alaska |
| 2013 | United States | 87 | 59.127 | -148.65 | Central Gulf of Alaska |
| 2013 | United States | 87 | 59.127 | -148.65 | Central Gulf of Alaska |
| 2013 | United States | 87 | 59.127 | -148.65 | Central Gulf of Alaska |
| 2013 | United States | 87 | 59.127 | -148.65 | Central Gulf of Alaska |
| 2013 | United States | 87 | 59.127 | -148.65 | Central Gulf of Alaska |
| 2013 | United States | 88 | 59.155 | -147.603 | Central Gulf of Alaska |
| 2013 | United States | 88 | 59.155 | -147.603 | Central Gulf of Alaska |
| 2013 | United States | 88 | 59.155 | -147.603 | Central Gulf of Alaska |
| 2013 | United States | 88 | 59.155 | -147.603 | Central Gulf of Alaska |
| 2013 | United States | 88 | 59.155 | -147.603 | Central Gulf of Alaska |
| 2013 | United States | 88 | 59.155 | -147.603 | Central Gulf of Alaska |
| 2013 | United States | 88 | 59.155 | -147.603 | Central Gulf of Alaska |
| 2013 | United States | 89 | 59.263 | -146.852 | West Yakutat |
| 2013 | United States | 89 | 59.263 | -146.852 | West Yakutat |
| 2013 | United States | 89 | 59.263 | -146.852 | West Yakutat |
| 2013 | United States | 89 | 59.263 | -146.852 | West Yakutat |
| 2013 | United States | 89 | 59.263 | -146.852 | West Yakutat |
| 2013 | United States | 89 | 59.263 | -146.852 | West Yakutat |
| 2013 | United States | 89 | 59.263 | -146.852 | West Yakutat |
| 2013 | United States | 90 | 59.5 | -145.525 | West Yakutat |
| 2013 | United States | 90 | 59.5 | -145.525 | West Yakutat |
| 2013 | United States | 90 | 59.5 | -145.525 | West Yakutat |
| 2013 | United States | 90 | 59.5 | -145.525 | West Yakutat |
| 2013 | United States | 90 | 59.5 | -145.525 | West Yakutat |
| 2013 | United States | 90 | 59.5 | -145.525 | West Yakutat |
| 2013 | United States | 90 | 59.5 | -145.525 | West Yakutat |
| 2013 | United States | 91 | 59.522 | -144.712 | West Yakutat |
| 2013 | United States | 91 | 59.522 | -144.712 | West Yakutat |
| 2013 | United States | 91 | 59.522 | -144.712 | West Yakutat |
| 2013 | United States | 91 | 59.522 | -144.712 | West Yakutat |
| 2013 | United States | 91 | 59.522 | -144.712 | West Yakutat |
| 2013 | United States | 91 | 59.522 | -144.712 | West Yakutat |
| 2013 | United States | 91 | 59.522 | -144.712 | West Yakutat |
| 2013 | United States | 92 | 59.555 | -143.652 | West Yakutat |
| 2013 | United States | 92 | 59.555 | -143.652 | West Yakutat |
| 2013 | United States | 92 | 59.555 | -143.652 | West Yakutat |
| 2013 | United States | 92 | 59.555 | -143.652 | West Yakutat |
| 2013 | United States | 92 | 59.555 | -143.652 | West Yakutat |
| 2013 | United States | 92 | 59.555 | -143.652 | West Yakutat |
| 2013 | United States | 92 | 59.555 | -143.652 | West Yakutat |
| 2013 | United States | 93 | 59.55 | -142.563 | West Yakutat |
| 2013 | United States | 93 | 59.55 | -142.563 | West Yakutat |
| 2013 | United States | 93 | 59.55 | -142.563 | West Yakutat |
| 2013 | United States | 93 | 59.55 | -142.563 | West Yakutat |
| 2013 | United States | 93 | 59.55 | -142.563 | West Yakutat |
| 2013 | United States | 93 | 59.55 | -142.563 | West Yakutat |
| 2013 | United States | 93 | 59.55 | -142.563 | West Yakutat |
| 2013 | United States | 94 | 59.388 | -142.163 | West Yakutat |
| 2013 | United States | 94 | 59.388 | -142.163 | West Yakutat |
| 2013 | United States | 94 | 59.388 | -142.163 | West Yakutat |
| 2013 | United States | 94 | 59.388 | -142.163 | West Yakutat |
| 2013 | United States | 94 | 59.388 | -142.163 | West Yakutat |
| 2013 | United States | 94 | 59.388 | -142.163 | West Yakutat |
| 2013 | United States | 95 | 59.05 | -141.343 | West Yakutat |
| 2013 | United States | 95 | 59.05 | -141.343 | West Yakutat |
| 2013 | United States | 95 | 59.05 | -141.343 | West Yakutat |
| 2013 | United States | 95 | 59.05 | -141.343 | West Yakutat |
| 2013 | United States | 95 | 59.05 | -141.343 | West Yakutat |
| 2013 | United States | 95 | 59.05 | -141.343 | West Yakutat |
| 2013 | United States | 96 | 58.685 | -140.64 | West Yakutat |
| 2013 | United States | 96 | 58.685 | -140.64 | West Yakutat |
| 2013 | United States | 96 | 58.685 | -140.64 | West Yakutat |
| 2013 | United States | 96 | 58.685 | -140.64 | West Yakutat |
| 2013 | United States | 96 | 58.685 | -140.64 | West Yakutat |
| 2013 | United States | 96 | 58.685 | -140.64 | West Yakutat |
| 2013 | United States | 97 | 58.468 | -139.467 | East Yakutat/Southeast |
| 2013 | United States | 97 | 58.468 | -139.467 | East Yakutat/Southeast |
| 2013 | United States | 97 | 58.468 | -139.467 | East Yakutat/Southeast |
| 2013 | United States | 97 | 58.468 | -139.467 | East Yakutat/Southeast |
| 2013 | United States | 97 | 58.468 | -139.467 | East Yakutat/Southeast |
| 2013 | United States | 97 | 58.468 | -139.467 | East Yakutat/Southeast |
| 2013 | United States | 97 | 58.468 | -139.467 | East Yakutat/Southeast |
| 2013 | United States | 98 | 58.14 | -138.73 | East Yakutat/Southeast |
| 2013 | United States | 98 | 58.14 | -138.73 | East Yakutat/Southeast |
| 2013 | United States | 98 | 58.14 | -138.73 | East Yakutat/Southeast |
| 2013 | United States | 98 | 58.14 | -138.73 | East Yakutat/Southeast |
| 2013 | United States | 98 | 58.14 | -138.73 | East Yakutat/Southeast |
| 2013 | United States | 98 | 58.14 | -138.73 | East Yakutat/Southeast |
| 2013 | United States | 99 | 57.878 | -137.378 | East Yakutat/Southeast |
| 2013 | United States | 99 | 57.878 | -137.378 | East Yakutat/Southeast |
| 2013 | United States | 99 | 57.878 | -137.378 | East Yakutat/Southeast |
| 2013 | United States | 99 | 57.878 | -137.378 | East Yakutat/Southeast |
| 2013 | United States | 99 | 57.878 | -137.378 | East Yakutat/Southeast |
| 2013 | United States | 99 | 57.878 | -137.378 | East Yakutat/Southeast |
| 2013 | United States | 100 | 57.618 | -136.537 | East Yakutat/Southeast |
| 2013 | United States | 100 | 57.618 | -136.537 | East Yakutat/Southeast |
| 2013 | United States | 100 | 57.618 | -136.537 | East Yakutat/Southeast |
| 2013 | United States | 100 | 57.618 | -136.537 | East Yakutat/Southeast |
| 2013 | United States | 100 | 57.618 | -136.537 | East Yakutat/Southeast |
| 2013 | United States | 100 | 57.618 | -136.537 | East Yakutat/Southeast |
| 2013 | United States | 101 | 57.188 | -136.235 | East Yakutat/Southeast |
| 2013 | United States | 101 | 57.188 | -136.235 | East Yakutat/Southeast |
| 2013 | United States | 101 | 57.188 | -136.235 | East Yakutat/Southeast |
| 2013 | United States | 101 | 57.188 | -136.235 | East Yakutat/Southeast |
| 2013 | United States | 101 | 57.188 | -136.235 | East Yakutat/Southeast |
| 2013 | United States | 101 | 57.188 | -136.235 | East Yakutat/Southeast |
| 2013 | United States | 101 | 57.188 | -136.235 | East Yakutat/Southeast |
| 2013 | United States | 102 | 56.852 | -135.997 | East Yakutat/Southeast |
| 2013 | United States | 102 | 56.852 | -135.997 | East Yakutat/Southeast |
| 2013 | United States | 102 | 56.852 | -135.997 | East Yakutat/Southeast |
| 2013 | United States | 102 | 56.852 | -135.997 | East Yakutat/Southeast |
| 2013 | United States | 102 | 56.852 | -135.997 | East Yakutat/Southeast |
| 2013 | United States | 102 | 56.852 | -135.997 | East Yakutat/Southeast |
| 2013 | United States | 102 | 56.852 | -135.997 | East Yakutat/Southeast |
| 2013 | United States | 103 | 56.383 | -135.348 | East Yakutat/Southeast |
| 2013 | United States | 103 | 56.383 | -135.348 | East Yakutat/Southeast |
| 2013 | United States | 103 | 56.383 | -135.348 | East Yakutat/Southeast |
| 2013 | United States | 103 | 56.383 | -135.348 | East Yakutat/Southeast |
| 2013 | United States | 104 | 55.983 | -135.437 | East Yakutat/Southeast |
| 2013 | United States | 104 | 55.983 | -135.437 | East Yakutat/Southeast |
| 2013 | United States | 104 | 55.983 | -135.437 | East Yakutat/Southeast |
| 2013 | United States | 104 | 55.983 | -135.437 | East Yakutat/Southeast |
| 2013 | United States | 104 | 55.983 | -135.437 | East Yakutat/Southeast |
| 2013 | United States | 104 | 55.983 | -135.437 | East Yakutat/Southeast |
| 2013 | United States | 105 | 55.558 | -134.967 | East Yakutat/Southeast |
| 2013 | United States | 105 | 55.558 | -134.967 | East Yakutat/Southeast |
| 2013 | United States | 105 | 55.558 | -134.967 | East Yakutat/Southeast |
| 2013 | United States | 105 | 55.558 | -134.967 | East Yakutat/Southeast |
| 2013 | United States | 105 | 55.558 | -134.967 | East Yakutat/Southeast |
| 2013 | United States | 105 | 55.558 | -134.967 | East Yakutat/Southeast |
| 2013 | United States | 105 | 55.558 | -134.967 | East Yakutat/Southeast |
| 2013 | United States | 106 | 55.347 | -134.735 | East Yakutat/Southeast |
| 2013 | United States | 106 | 55.347 | -134.735 | East Yakutat/Southeast |
| 2013 | United States | 106 | 55.347 | -134.735 | East Yakutat/Southeast |
| 2013 | United States | 106 | 55.347 | -134.735 | East Yakutat/Southeast |
| 2013 | United States | 106 | 55.347 | -134.735 | East Yakutat/Southeast |
| 2013 | United States | 106 | 55.347 | -134.735 | East Yakutat/Southeast |
| 2013 | United States | 107 | 54.9 | -134.287 | East Yakutat/Southeast |
| 2013 | United States | 107 | 54.9 | -134.287 | East Yakutat/Southeast |
| 2013 | United States | 107 | 54.9 | -134.287 | East Yakutat/Southeast |
| 2013 | United States | 107 | 54.9 | -134.287 | East Yakutat/Southeast |
| 2013 | United States | 107 | 54.9 | -134.287 | East Yakutat/Southeast |
| 2013 | United States | 107 | 54.9 | -134.287 | East Yakutat/Southeast |
| 2013 | United States | 107 | 54.9 | -134.287 | East Yakutat/Southeast |
| 2013 | United States | 108 | 54.45 | -133.93 | East Yakutat/Southeast |
| 2013 | United States | 108 | 54.45 | -133.93 | East Yakutat/Southeast |
| 2013 | United States | 108 | 54.45 | -133.93 | East Yakutat/Southeast |
| 2013 | United States | 108 | 54.45 | -133.93 | East Yakutat/Southeast |
| 2013 | United States | 108 | 54.45 | -133.93 | East Yakutat/Southeast |
| 2013 | United States | 108 | 54.45 | -133.93 | East Yakutat/Southeast |
| 2013 | United States | 108 | 54.45 | -133.93 | East Yakutat/Southeast |
| 2013 | United States | 120 | 55.788 | -156.077 | Central Gulf of Alaska |
| 2013 | United States | 120 | 55.788 | -156.077 | Central Gulf of Alaska |
| 2013 | United States | 120 | 55.788 | -156.077 | Central Gulf of Alaska |
| 2013 | United States | 120 | 55.788 | -156.077 | Central Gulf of Alaska |
| 2013 | United States | 121 | 55.75 | -156.202 | Central Gulf of Alaska |
| 2013 | United States | 121 | 55.75 | -156.202 | Central Gulf of Alaska |
| 2013 | United States | 121 | 55.75 | -156.202 | Central Gulf of Alaska |
| 2013 | United States | 121 | 55.75 | -156.202 | Central Gulf of Alaska |
| 2013 | United States | 122 | 56.187 | -155.963 | Central Gulf of Alaska |
| 2013 | United States | 122 | 56.187 | -155.963 | Central Gulf of Alaska |
| 2013 | United States | 122 | 56.187 | -155.963 | Central Gulf of Alaska |
| 2013 | United States | 122 | 56.187 | -155.963 | Central Gulf of Alaska |
| 2013 | United States | 123 | 56.232 | -156.13 | Central Gulf of Alaska |
| 2013 | United States | 123 | 56.232 | -156.13 | Central Gulf of Alaska |
| 2013 | United States | 123 | 56.232 | -156.13 | Central Gulf of Alaska |
| 2013 | United States | 123 | 56.232 | -156.13 | Central Gulf of Alaska |
| 2013 | United States | 124 | 56.988 | -155.063 | Central Gulf of Alaska |
| 2013 | United States | 124 | 56.988 | -155.063 | Central Gulf of Alaska |
| 2013 | United States | 124 | 56.988 | -155.063 | Central Gulf of Alaska |
| 2013 | United States | 125 | 57.002 | -155.303 | Central Gulf of Alaska |
| 2013 | United States | 125 | 57.002 | -155.303 | Central Gulf of Alaska |
| 2013 | United States | 125 | 57.002 | -155.303 | Central Gulf of Alaska |
| 2013 | United States | 126 | 57.347 | -155.04 | Central Gulf of Alaska |
| 2013 | United States | 126 | 57.347 | -155.04 | Central Gulf of Alaska |
| 2013 | United States | 126 | 57.347 | -155.04 | Central Gulf of Alaska |
| 2013 | United States | 127 | 57.348 | -155.245 | Central Gulf of Alaska |
| 2013 | United States | 127 | 57.348 | -155.245 | Central Gulf of Alaska |
| 2013 | United States | 127 | 57.348 | -155.245 | Central Gulf of Alaska |
| 2013 | United States | 128 | 58 | -149.842 | Central Gulf of Alaska |
| 2013 | United States | 128 | 58 | -149.842 | Central Gulf of Alaska |
| 2013 | United States | 128 | 58 | -149.842 | Central Gulf of Alaska |
| 2013 | United States | 128 | 58 | -149.842 | Central Gulf of Alaska |
| 2013 | United States | 128 | 58 | -149.842 | Central Gulf of Alaska |
| 2013 | United States | 128 | 58 | -149.842 | Central Gulf of Alaska |
| 2013 | United States | 129 | 58.083 | -149.908 | Central Gulf of Alaska |
| 2013 | United States | 129 | 58.083 | -149.908 | Central Gulf of Alaska |
| 2013 | United States | 129 | 58.083 | -149.908 | Central Gulf of Alaska |
| 2013 | United States | 129 | 58.083 | -149.908 | Central Gulf of Alaska |
| 2013 | United States | 129 | 58.083 | -149.908 | Central Gulf of Alaska |
| 2013 | United States | 130 | 58.727 | -149.197 | Central Gulf of Alaska |
| 2013 | United States | 130 | 58.727 | -149.197 | Central Gulf of Alaska |
| 2013 | United States | 130 | 58.727 | -149.197 | Central Gulf of Alaska |
| 2013 | United States | 130 | 58.727 | -149.197 | Central Gulf of Alaska |
| 2013 | United States | 130 | 58.727 | -149.197 | Central Gulf of Alaska |
| 2013 | United States | 130 | 58.727 | -149.197 | Central Gulf of Alaska |
| 2013 | United States | 131 | 58.802 | -149.048 | Central Gulf of Alaska |
| 2013 | United States | 131 | 58.802 | -149.048 | Central Gulf of Alaska |
| 2013 | United States | 131 | 58.802 | -149.048 | Central Gulf of Alaska |
| 2013 | United States | 131 | 58.802 | -149.048 | Central Gulf of Alaska |
| 2013 | United States | 131 | 58.802 | -149.048 | Central Gulf of Alaska |
| 2013 | United States | 131 | 58.802 | -149.048 | Central Gulf of Alaska |
| 2013 | United States | 132 | 59.083 | -149.4 | Central Gulf of Alaska |
| 2013 | United States | 132 | 59.083 | -149.4 | Central Gulf of Alaska |
| 2013 | United States | 132 | 59.083 | -149.4 | Central Gulf of Alaska |
| 2013 | United States | 132 | 59.083 | -149.4 | Central Gulf of Alaska |
| 2013 | United States | 133 | 58.95 | -149.508 | Central Gulf of Alaska |
| 2013 | United States | 133 | 58.95 | -149.508 | Central Gulf of Alaska |
| 2013 | United States | 133 | 58.95 | -149.508 | Central Gulf of Alaska |
| 2013 | United States | 133 | 58.95 | -149.508 | Central Gulf of Alaska |
| 2013 | United States | 133 | 58.95 | -149.508 | Central Gulf of Alaska |
| 2013 | United States | 134 | 59.615 | -146.967 | Central Gulf of Alaska |
| 2013 | United States | 134 | 59.615 | -146.967 | Central Gulf of Alaska |
| 2013 | United States | 134 | 59.615 | -146.967 | Central Gulf of Alaska |
| 2013 | United States | 134 | 59.615 | -146.967 | Central Gulf of Alaska |
| 2013 | United States | 134 | 59.615 | -146.967 | Central Gulf of Alaska |
| 2013 | United States | 135 | 59.515 | -147.153 | Central Gulf of Alaska |
| 2013 | United States | 135 | 59.515 | -147.153 | Central Gulf of Alaska |
| 2013 | United States | 135 | 59.515 | -147.153 | Central Gulf of Alaska |
| 2013 | United States | 135 | 59.515 | -147.153 | Central Gulf of Alaska |
| 2013 | United States | 135 | 59.515 | -147.153 | Central Gulf of Alaska |
| 2013 | United States | 136 | 59.747 | -143.587 | West Yakutat |
| 2013 | United States | 136 | 59.747 | -143.587 | West Yakutat |
| 2013 | United States | 136 | 59.747 | -143.587 | West Yakutat |
| 2013 | United States | 136 | 59.747 | -143.587 | West Yakutat |
| 2013 | United States | 136 | 59.747 | -143.587 | West Yakutat |
| 2013 | United States | 137 | 59.673 | -143.382 | West Yakutat |
| 2013 | United States | 137 | 59.673 | -143.382 | West Yakutat |
| 2013 | United States | 137 | 59.673 | -143.382 | West Yakutat |
| 2013 | United States | 137 | 59.673 | -143.382 | West Yakutat |
| 2013 | United States | 137 | 59.673 | -143.382 | West Yakutat |
| 2013 | United States | 137 | 59.673 | -143.382 | West Yakutat |
| 2013 | United States | 138 | 59.417 | -140.937 | West Yakutat |
| 2013 | United States | 138 | 59.417 | -140.937 | West Yakutat |
| 2013 | United States | 138 | 59.417 | -140.937 | West Yakutat |
| 2013 | United States | 138 | 59.417 | -140.937 | West Yakutat |
| 2013 | United States | 138 | 59.417 | -140.937 | West Yakutat |
| 2013 | United States | 139 | 59.413 | -141.168 | West Yakutat |
| 2013 | United States | 139 | 59.413 | -141.168 | West Yakutat |
| 2013 | United States | 139 | 59.413 | -141.168 | West Yakutat |
| 2013 | United States | 139 | 59.413 | -141.168 | West Yakutat |
| 2013 | United States | 139 | 59.413 | -141.168 | West Yakutat |
| 2013 | United States | 142 | 57.915 | -137.01 | East Yakutat/Southeast |
| 2013 | United States | 142 | 57.915 | -137.01 | East Yakutat/Southeast |
| 2013 | United States | 142 | 57.915 | -137.01 | East Yakutat/Southeast |
| 2013 | United States | 142 | 57.915 | -137.01 | East Yakutat/Southeast |
| 2013 | United States | 142 | 57.915 | -137.01 | East Yakutat/Southeast |
| 2013 | United States | 142 | 57.915 | -137.01 | East Yakutat/Southeast |
| 2013 | United States | 143 | 57.967 | -137.077 | East Yakutat/Southeast |
| 2013 | United States | 143 | 57.967 | -137.077 | East Yakutat/Southeast |
| 2013 | United States | 143 | 57.967 | -137.077 | East Yakutat/Southeast |
| 2013 | United States | 143 | 57.967 | -137.077 | East Yakutat/Southeast |
| 2013 | United States | 143 | 57.967 | -137.077 | East Yakutat/Southeast |
| 2013 | United States | 143 | 57.967 | -137.077 | East Yakutat/Southeast |
| 2013 | United States | 144 | 55.93 | -134.902 | East Yakutat/Southeast |
| 2013 | United States | 144 | 55.93 | -134.902 | East Yakutat/Southeast |
| 2013 | United States | 144 | 55.93 | -134.902 | East Yakutat/Southeast |
| 2013 | United States | 144 | 55.93 | -134.902 | East Yakutat/Southeast |
| 2013 | United States | 144 | 55.93 | -134.902 | East Yakutat/Southeast |
| 2013 | United States | 144 | 55.93 | -134.902 | East Yakutat/Southeast |
| 2013 | United States | 145 | 56.033 | -134.927 | East Yakutat/Southeast |
| 2013 | United States | 145 | 56.033 | -134.927 | East Yakutat/Southeast |
| 2013 | United States | 145 | 56.033 | -134.927 | East Yakutat/Southeast |
| 2013 | United States | 145 | 56.033 | -134.927 | East Yakutat/Southeast |
| 2013 | United States | 145 | 56.033 | -134.927 | East Yakutat/Southeast |
| 2013 | United States | 148 | 54.648 | -132.838 | East Yakutat/Southeast |
| 2013 | United States | 148 | 54.648 | -132.838 | East Yakutat/Southeast |
| 2013 | United States | 148 | 54.648 | -132.838 | East Yakutat/Southeast |
| 2013 | United States | 148 | 54.648 | -132.838 | East Yakutat/Southeast |
| 2013 | United States | 148 | 54.648 | -132.838 | East Yakutat/Southeast |
| 2013 | United States | 148 | 54.648 | -132.838 | East Yakutat/Southeast |
| 2013 | United States | 149 | 54.598 | -133.023 | East Yakutat/Southeast |
| 2013 | United States | 149 | 54.598 | -133.023 | East Yakutat/Southeast |
| 2013 | United States | 149 | 54.598 | -133.023 | East Yakutat/Southeast |
| 2013 | United States | 149 | 54.598 | -133.023 | East Yakutat/Southeast |
| 2013 | United States | 149 | 54.598 | -133.023 | East Yakutat/Southeast |
| 2013 | United States | 149 | 54.598 | -133.023 | East Yakutat/Southeast |

Figure S1: Cumulative number of stations fit with linear, exponential, logarithmic, and sigmoid functions.


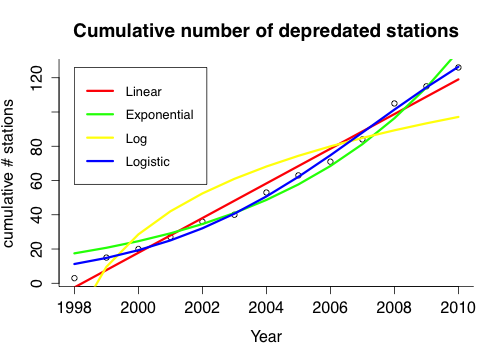


Table S2: Stations with depredation, 1998-2010. Station numbers are given along with the number of individuals observed at those stations (in parenthesis) for the years in which these data were recorded.

| 1998 | 89, 90, 94, 96 |
| --- | --- |
| 1999 | 82, 85, 88, 89, 91, 92, 93, 95, 98, 100, 101, 102 |
| 2000 | 92, 95, 97, 102, 103 |
| 2001 | 83, 84, 85, 99, 100, 138, 139 |
| 2002 | 84, 86, 88, 89, 93, 94, 95, 97, 98 |
| 2003 | 88, 95, 99, 104 |
| 2004 | 85, 86, 88, 89, 90, 93, 94, 98, 99, 100, 101, 104, 108 |
| 2005 | 89, 93, 97, 100(3), 101(3), 102(3),104, 105, 106, 143 |
| 2006 | 86, 88, 89, 90, 94, 95, 99, 101 |
| 2007 | 86, 88(3), 89, 90, 91, 92 , 93, 100(3), 101(3), 102(3), 104(2), 105, 106 |
| 2008 | 86(4), 87(4), 88(6), 89(1), 90(2), 91(7), 92(4), 93(6), 94(6), 95(4), 96, 97, 100(6), 101(2), 102(3), 103(5), 104(4), 105, 106(2), 107(2), 108(2) |
| 2009 | 72(3), 78(1), 84(4), 85(2), 86(2), 89(6), 95(3), 96(2), 99, 100(3) |
| 2010 | 82(3), 86(2), 94(4), 96(3), 97(1), 101(2), 102(2), 104(1), 105(2), 106(1), 108(2) |
